# Supplementary material for: TRIM66 reads unmodified H3R2K4 and H3K56ac to respond to DNA damage in embryonic stem cells
Source: Nat Commun. 2019 Sep 19;10:4273. doi: 10.1038/s41467-019-12126-4 (PMC6753139; doi:10.1038/s41467-019-12126-4)
Supplement: Supplementary file 1 — Supplementary Information [file 41467_2019_12126_MOESM1_ESM.pdf]

Supplementary Information

TRIM66 reads unmodified H3R2K4 and H3K56ac to respond to DNA damage in embryonic stem cells

Chen et al.

Supplementary Figures: 1 – 14

Supplementary Tables: 1 – 6

Supplementary Notes: 1 – 3

Supplementary Methods

Supplementary References

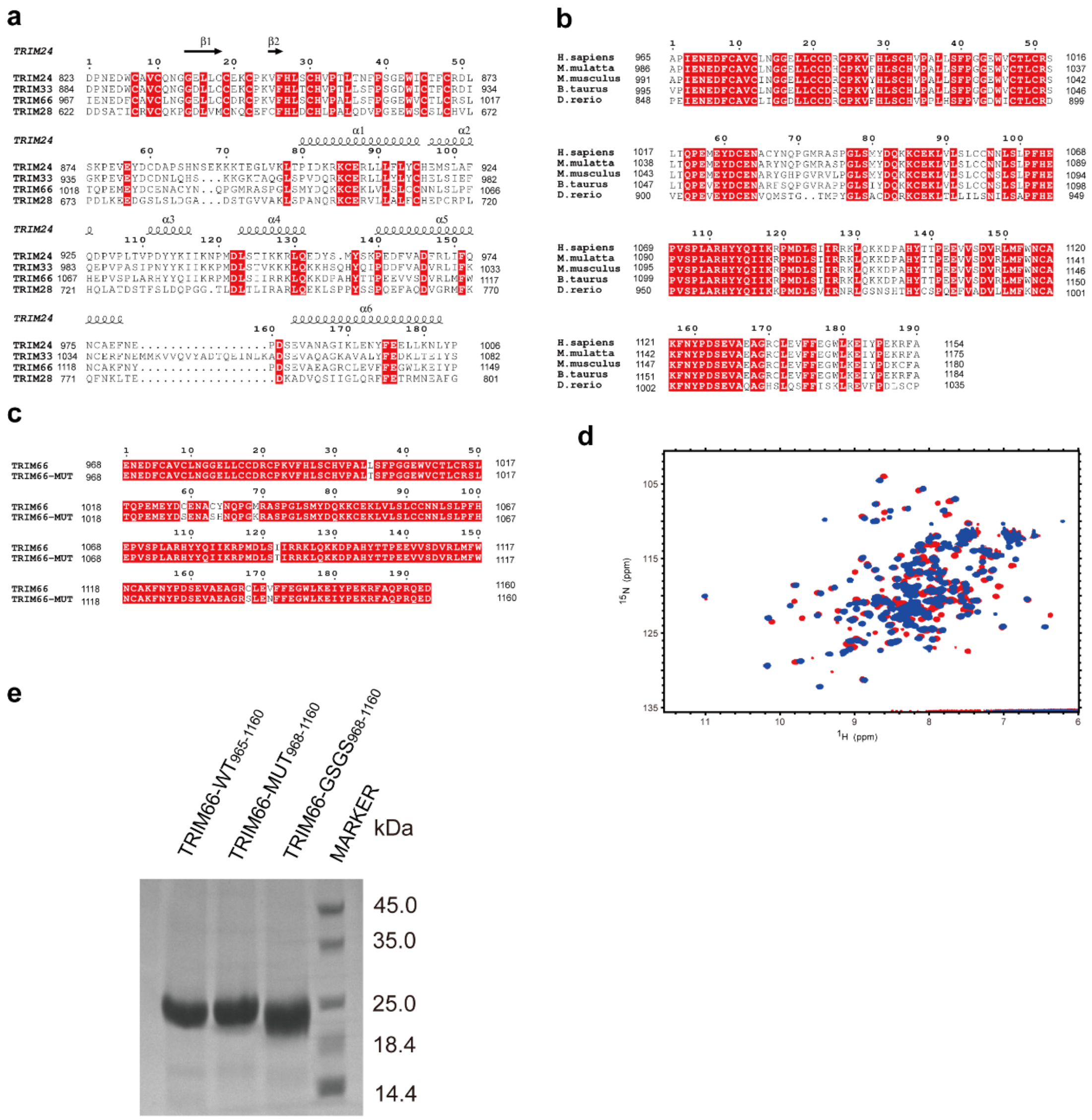

Supplementary Figure 1. The sequence comparison of human TRIM66 and some experimental results of TRIM66 PHD-Bromodomain proteins.

a, Sequence alignment of human TRIM66 PHD-Bromodomain and its paralogs. Conserved amino acid residues are shaded with a red background. The secondary structure of TRIM24-PHD-Bromo is shown with the according sequence. The α-helix (α) is shown as spiral line; the β-sheet (β) is shown as arrow line. **b**, Sequence alignment of the PHD-Bromodomain of TRIM66 from diverse species. Conserved amino acid residues are shaded with a red background. **c**, The sequence alignment of TRIM66-WT$_{968\text{-}1160}$ and TRIM66-MUT$_{968\text{-}1160}$. The alignment was generated by ESPript 3 with CLUSTALW. The aligned sequences are labeled with actual residual numbers. **d**, Superposition of ^{15}N–^{1}H HSQC spectra of TRIM66-WT$_{968\text{-}1160}$ (red) and TRIM66-MUT$_{968\text{-}1160}$ with eight mutated residues (L1002T, C1026S, C1030S, Y1031H, M1036K, I1089T, C1135S, and V1138N) (blue). **e**, TRIM66-WT$_{965\text{-}1160}$, TRIM66-MUT$_{968\text{-}1160}$ and TRIM66-GSGS$_{968\text{-}1160}$ proteins were analyzed by SDS-PAGE followed by Colloidal Blue staining. Source data are provided as a Source Data file.

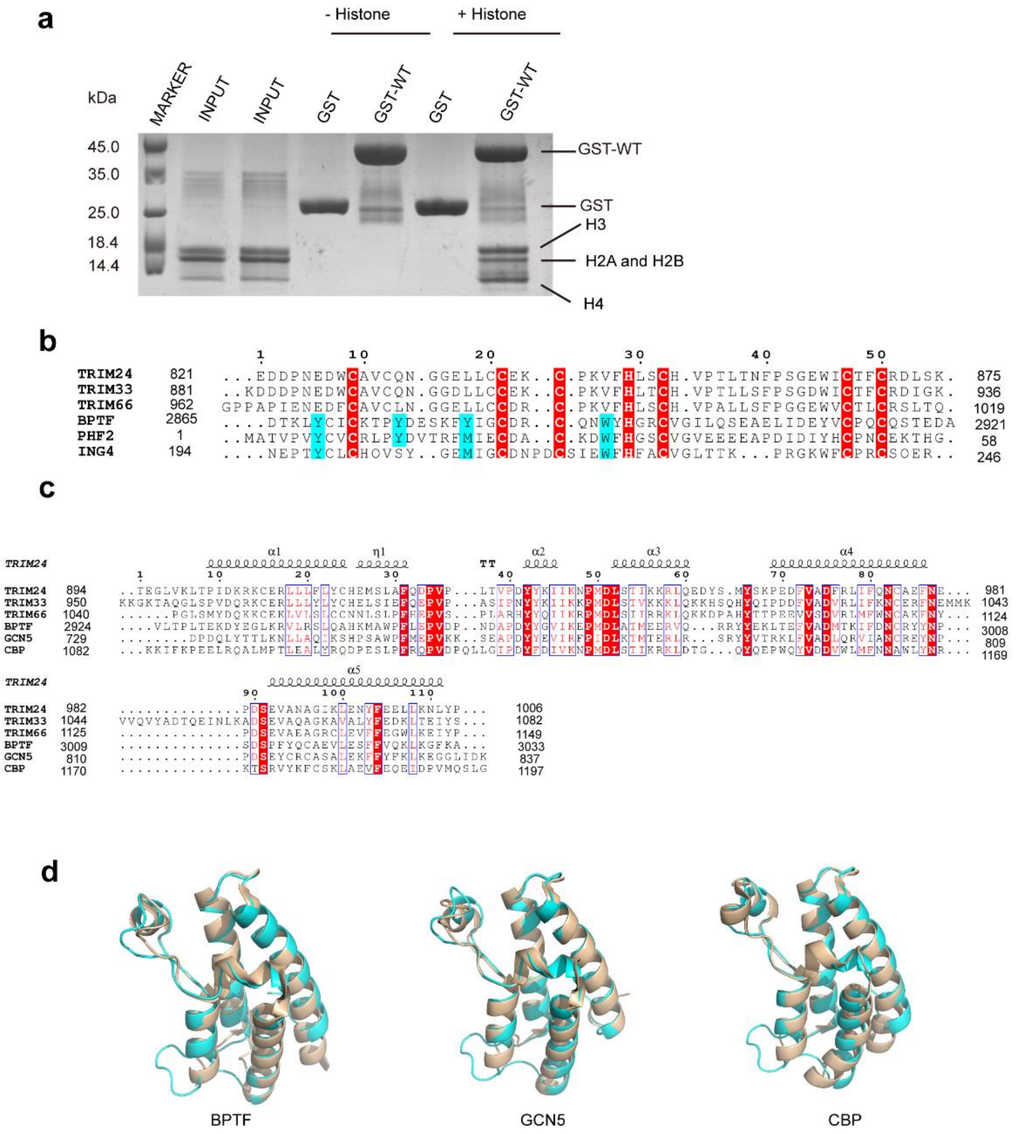

Supplementary Figure 2. The PHD-Bromodomain of TRIM66 is evolutionarily and structurally conserved module.

a, The GST pulldown assays of the TRIM66-WT$_{965\text{-}1160}$ against the calf thymus histones. Source data are provided as a Source Data file. **b,** Sequence alignment of PHDs of human TRIM24, TRIM33, TRIM66, BPTF, PHF2, and ING4. Amino acid residues shaded with a blue background are necessary for reading H3K4me2/3. **c,** Sequence alignment of Bromodomains of human TRIM24, TRIM33, TRIM66, BPTF, GCN5, and CBP. All the alignments are generated by ESPript 3 with CLUSTALW. Conserved amino acid residues are with a red background or in blue box. The aligned sequences are labeled with actual residual numbers. **d,** Structure comparison of Bromodomain of TRIM66 (in cyan) with BPTF (PDB ID:3UV2), GCN5 (PDB ID:3D7C), and CBP (PDB ID:3DWY) (in wheat). The structures are shown in cartoon.

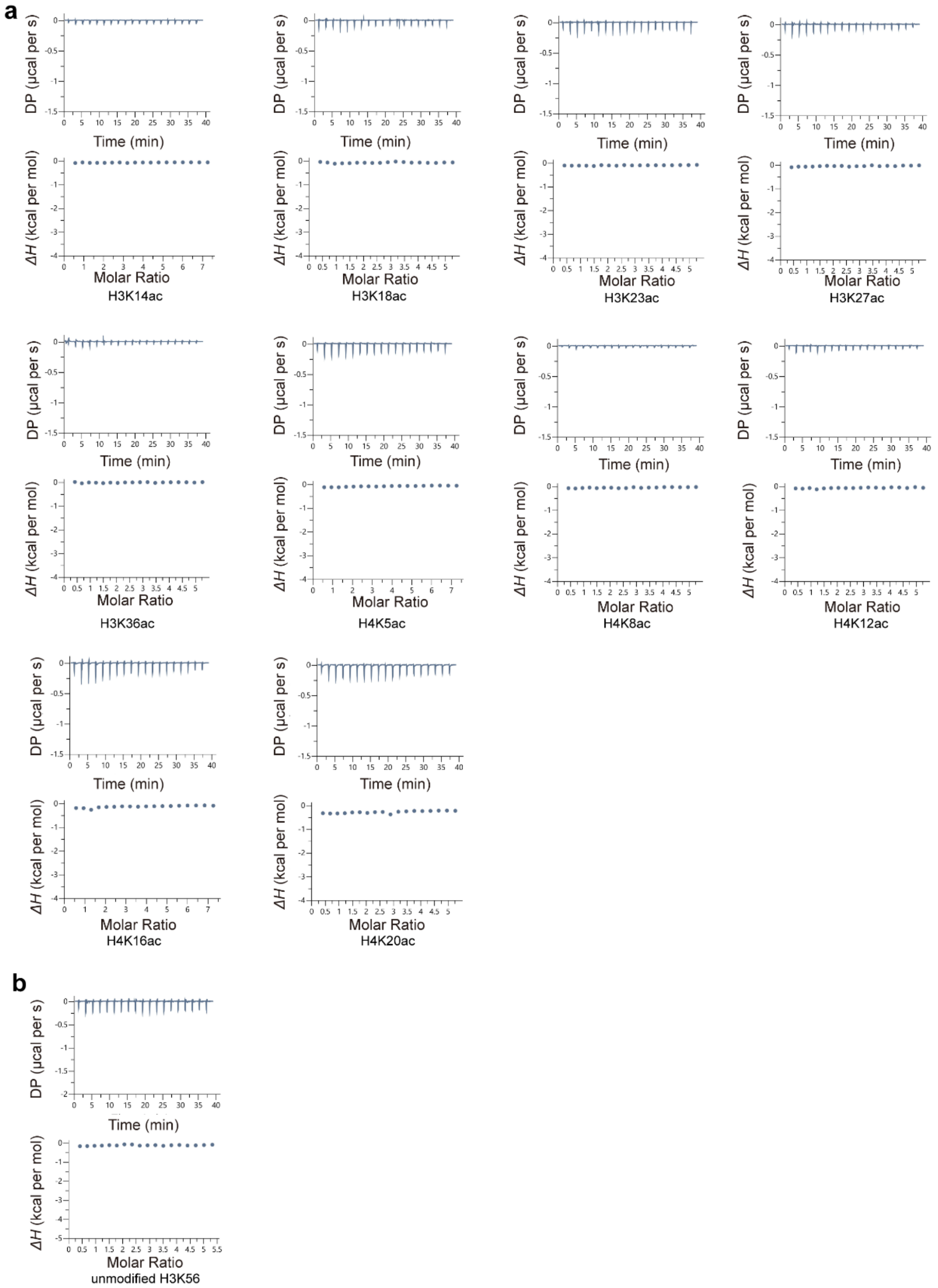

Supplementary Figure 3. Titrating TRIM66-WT$_{965\text{-}1160}$ proteins with different peptides derived from H3 and H4.

a, ITC titration: titrating TRIM66-WT$_{965\text{-}1160}$ with acetyl-lysine peptides derived from H3 and H4. **b,** ITC titration: titrating TRIM66-WT$_{965\text{-}1160}$ with unmodified H3K56 peptide.

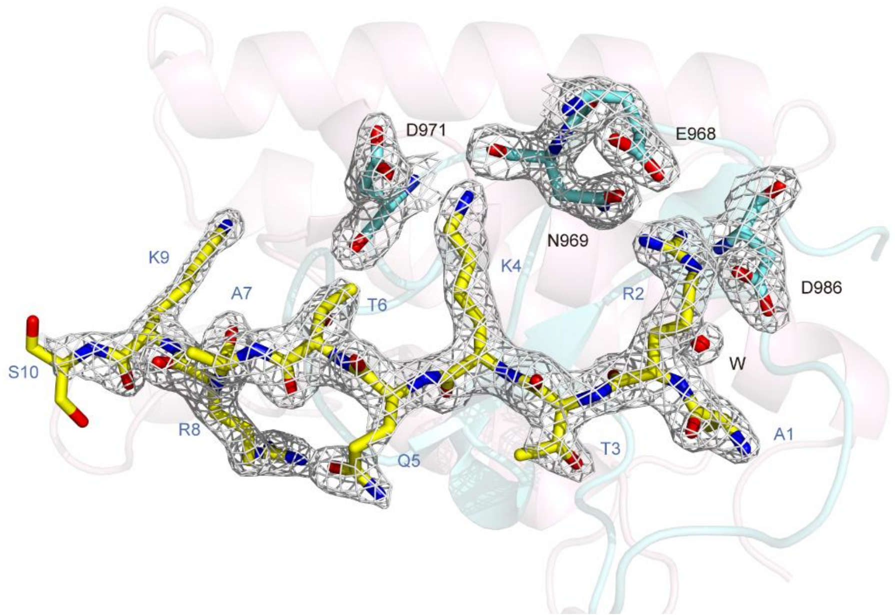

Supplementary Figure 4. Stereo view of a portion of the electron density map for TRIM66-MUT$_{968\text{-}1160}$-H3$_{1\text{-}12}$.

A portion of the 2Fo-Fc electron density (white mesh) for TRIM66-MUT$_{968\text{-}1160}$-H3$_{1\text{-}12}$ contoured at 1.2 σ is shown. The PHD finger domain (in aquamarine) and Bromodomain (in light pink) are shown as cartoon. Residues E968, N969, D971, D986 of TRIM66 and A1-S10 of H3 peptide (in yellow) are labeled and shown as sticks with red oxygen atoms and blue nitrogen atoms. A water molecule mediating the H3-TRIM66 PHD interaction is shown as a small red sphere.

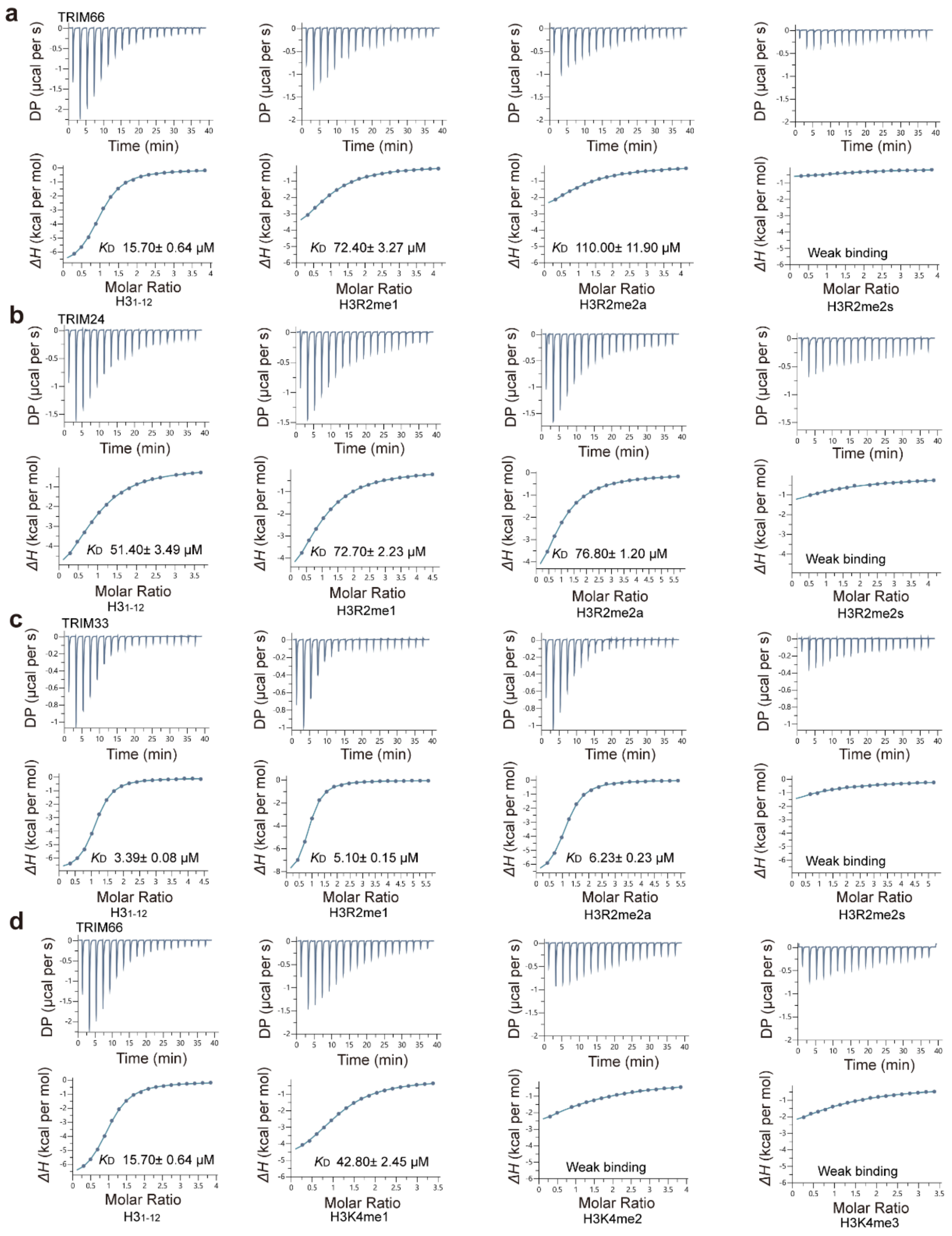

Supplementary Figure 5. Titrating TRIM66-WT$_{965\text{-}1160}$, TRIM24$_{824\text{-}1006}$, and TRIM33$_{882\text{-}1087}$ proteins with different methylated H3 peptides.

a, ITC titration: titrating TRIM66-WT$_{965\text{-}1160}$ with different methylated H3R2 peptides. **b,** ITC titration: titrating TRIM24$_{824\text{-}1006}$ with different methylated H3R2 peptides. **c,** ITC titration: titrating TRIM33$_{882\text{-}1087}$ with different methylated H3R2 peptides. **d,** ITC titration: titrating TRIM66-WT$_{965\text{-}1160}$ with different methylated H3K4 peptides.

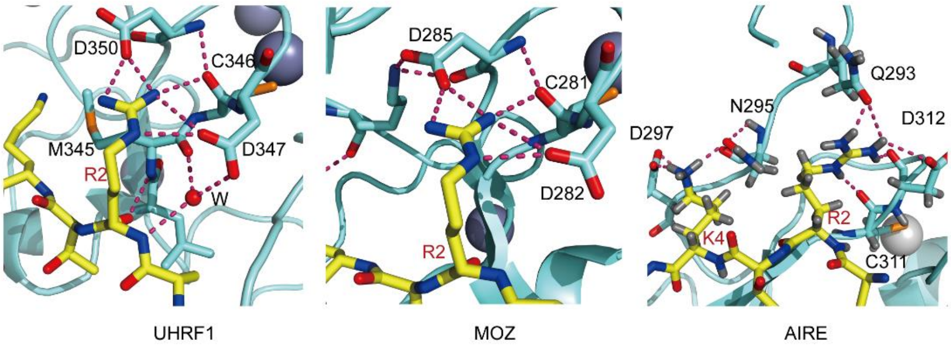

Supplementary Figure 6. Detailed interactions between H3R2 residue and PHDs from various proteins.

The key residues in the interaction between H3R2 residue (in yellow) and PHDs (in aquamarine) are labeled and shown in sticks with grey hydrogen atom, red oxygen atoms, blue nitrogen atoms, and orange sulfur atoms. The hydrogen bonds of the H3R2-PHD interaction are shown in warm pink dashed lines. The water molecules mediating the H3R2-PHD interaction are shown as a small red sphere. The zinc ions of PHDs are shown as sliver spheres. UHRF1, PDB code: 3SOU; MOZ, PDB code: 3V43; AIRE, PDB code: 2KFT.

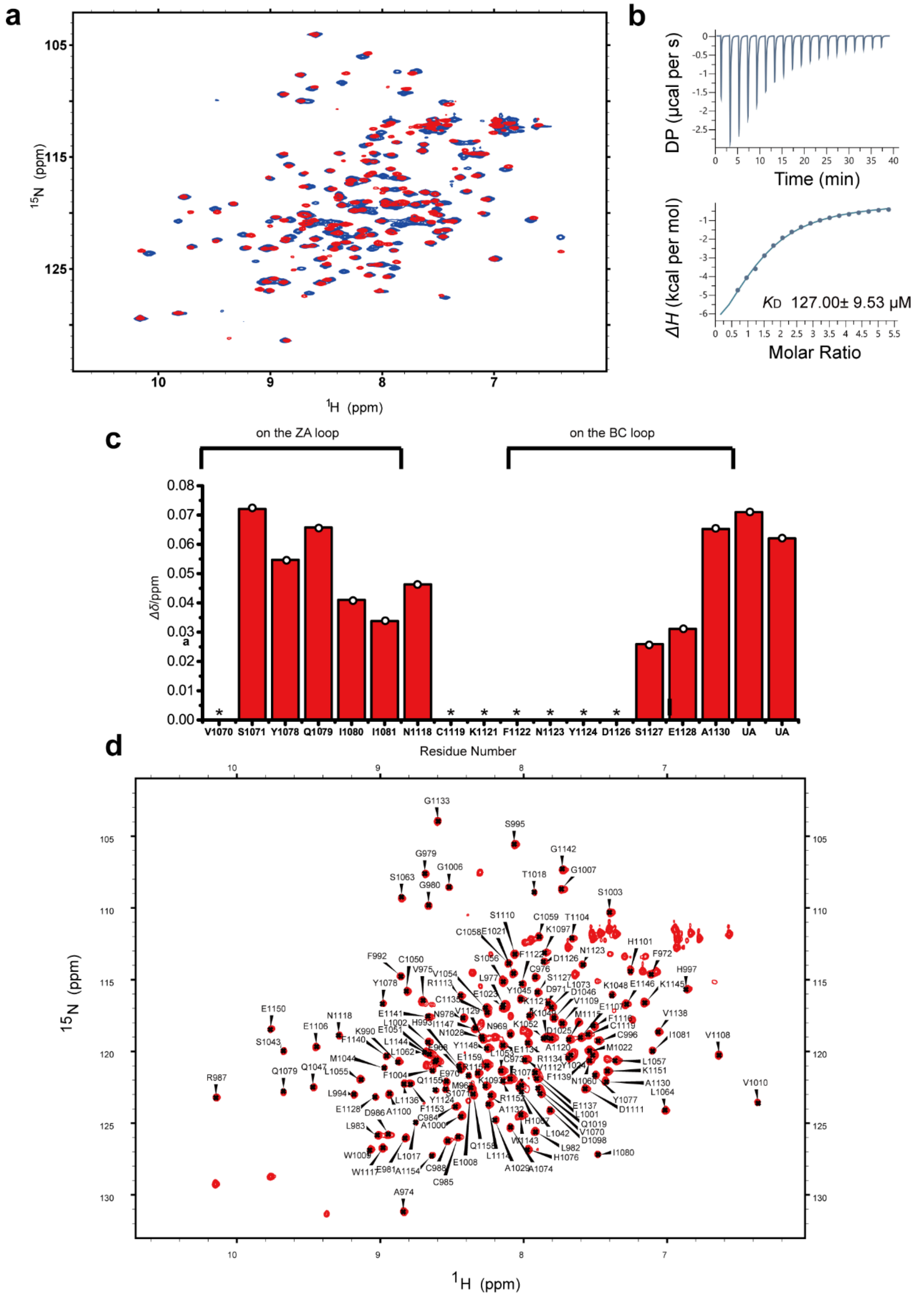

Supplementary Figure 7. Experimental characterization and sequential backbone assignment of TRIM66-GSGS$_{968\text{-}1160}$.

a, Superposition of ^{15}N–^{1}H HSQC spectra of TRIM66-WT$_{968\text{-}1160}$ (blue) and TRIM66-GSGS$_{968\text{-}1160}$ (red). **b**, ITC titration: titrating TRIM66-GSGS$_{968\text{-}1160}$ proteins with H3K56ac peptides. **c**, Quantification of chemical shift perturbations when titrating TRIM66-GSGS$_{968\text{-}1160}$ proteins with H3K56ac peptides. Histogram plot presents chemical shift perturbations upon the addition of H3K56ac peptide. The molar ratio of peptide/protein is 2.8:1. The

perturbation values were calculated as previous work[1]. The perturbed residues are denoted on the graph. The residues whose peak disappeared after titration are marked with *star*. *UA* presents residues which are unassigned. **d**, The backbone assignment of TRIM66-GSGS$_{968-1160}$ proteins. The corresponding residues are labeled.

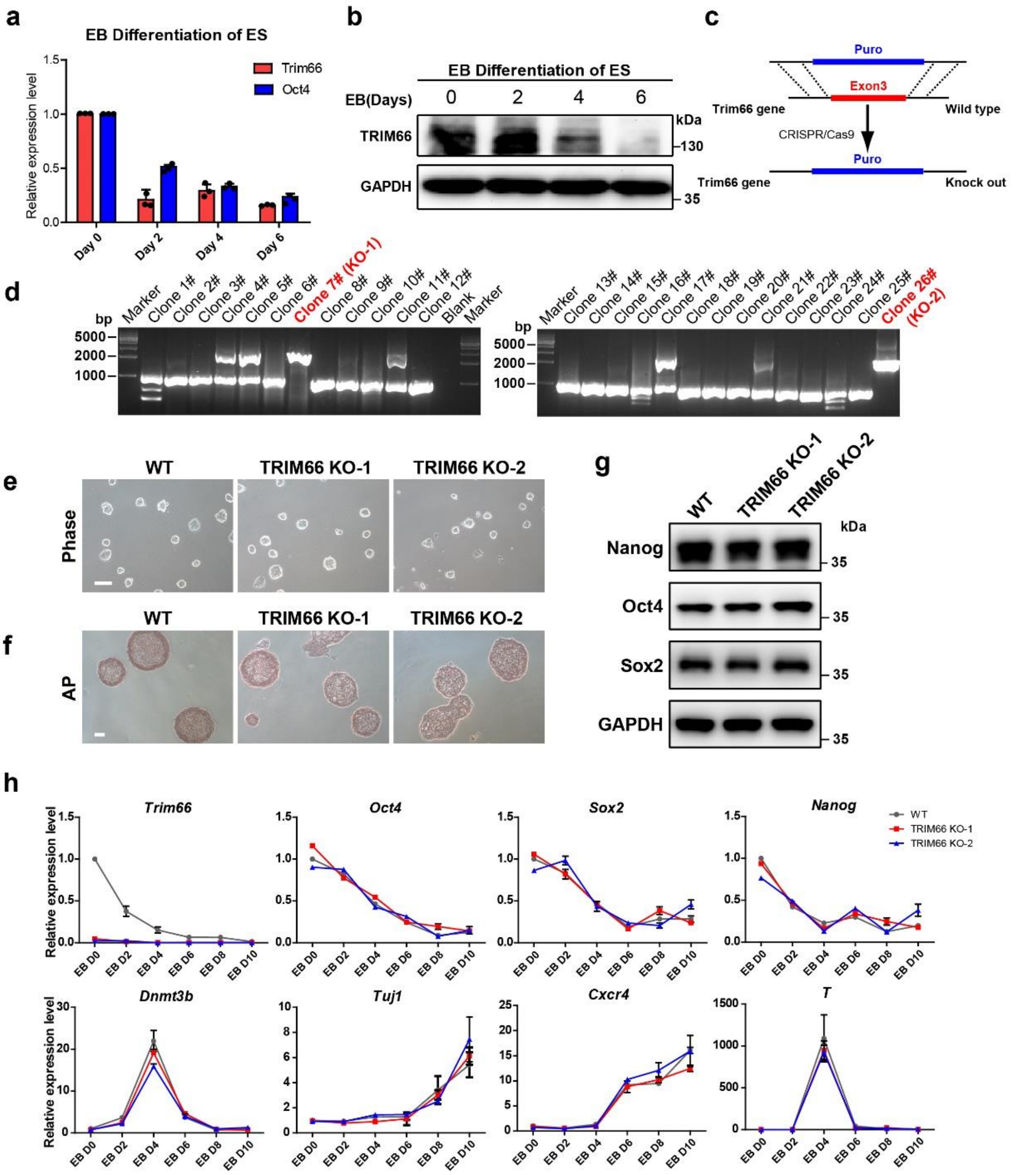

Supplementary Figure 8. Expression of Trim66 during ESC differentiation and the self-renewal analysis in the constructed Trim66 double knock-out ESC lines.

a, Expression levels of *Trim66* and *Oct4* determined by RT-qPCR analysis during EB differentiation. **b**, Immunoblot analysis of TRIM66 during EB differentiation. **c**, Schematic illustration of the establishment of TRIM66 KO ESC lines. **d**, PCR genotyping analysis of ESC lines. Clone 7# and clone 26# are determined as the Trim66 double knockout ESC lines, and termed as TRIM66 KO-1 and TRIM66 KO-2 ESC lines, respectively. **e, f,** Representative images of ESC morphology in **e** and alkaline phosphatase activity in **f** of WT and TRIM66 KO ESCs. Scale bar, 100 µm. **g,** Immunoblot analysis of pluripotency markers (Nanog, Oct4, and Sox2) in TRIM66 KO ESCs. **h,** The expression level of pluripotency markers (*Oct4, Sox2, and Nanog*) and the developmental genes (*Dnmt3b, Tuj1, Cxcr4, and T*) during EB differentiation of WT and TRIM66 KO ESCs is determined by RT-qPCR analysis. Data are presented as the means ± SEM. Source data are provided as a Source Data file.

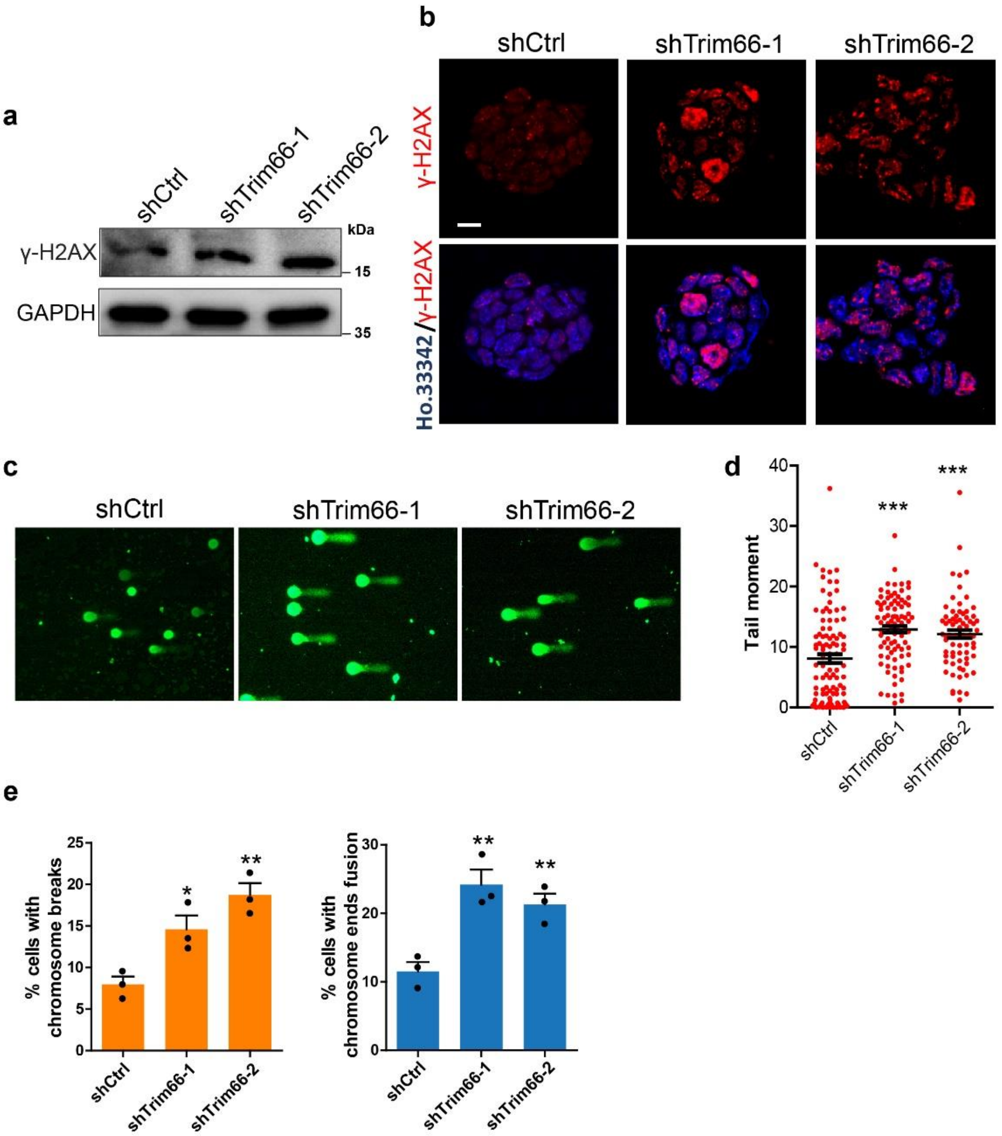

Supplementary Figure 9. Knockdown of Trim66 in ESCs with distinct genetic background generates similar phenotypes.

a, Immunoblot showing the increased γ-H2AX levels in shCtrl and shTrim66 ESCs. **b**, Representative immunofluorescence images show the formation of γ-H2AX foci in shCtrl and shTrim66 ESCs. Scale bar, 10 μm. **c**, Representative comet assay images of shCtrl and shTrim66 ESCs. **d**, DNA integrity assessment of shCtrl and shTrim66 ESCs by comet assay. More than 100 cells are examined in each sample. **e**, Quantification of chromosomal breakage (left) or chromosome ends fusion (right) in shCtrl and shTrim66 ESCs. More than 200 cells are examined in each sample. Data are presented as the means ± SEM. Statistical significance is determined by two-tailed Student's t test. $^*p < 0.05$, $^{**}p < 0.01$, $^{***}p < 0.001$. Source data are provided as a Source Data file.

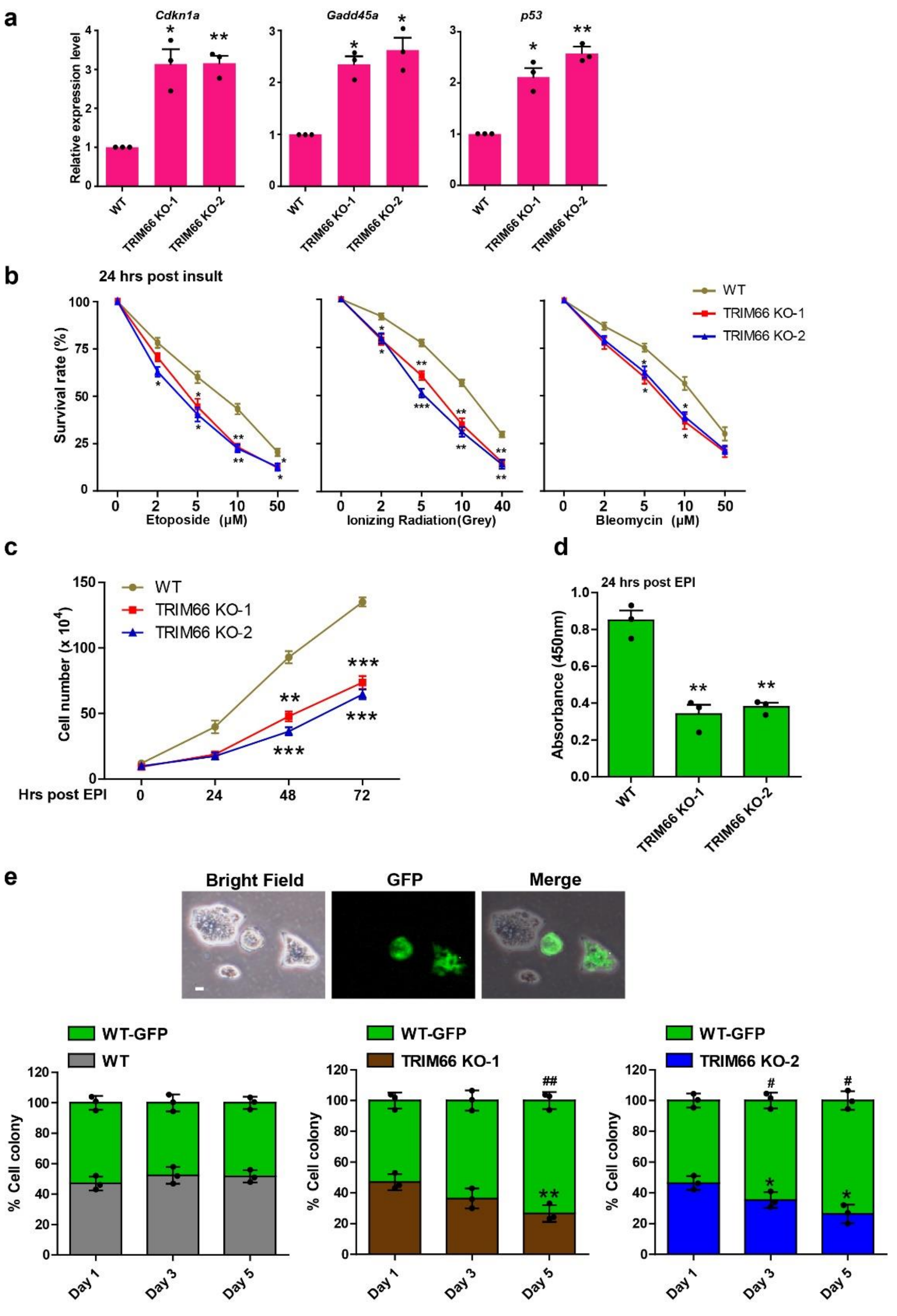

Supplementary Figure 10. TRIM66 is required for ESC to survive DNA damage.

a, The expression level of DNA damage related genes (*Cdkn1a*, *Gadd45a*, and *p53*) in WT and TRIM66 KO ESCs is determined by RT-qPCR analysis. **b,** Survival analysis of WT and TRIM66 KO ESCs treated with etoposide, ionizing radiation or bleomycin in different doses. **c, d,** Detection of cell growth through cell counting **b** and CCK8 assay **c** in WT and TRIM66 KO ESCs treated with EPI. **e,** Clonal competition assay of WT and TRIM66 KO ESCs treated with EPI in different days (day 1, 3, and 5). Representative images show the mixtures of WT-GFP and WT ESC clones. Scale bar, 10 μm.* represents a difference from WT ESCs or GFP-negative ESC clones at day 1, # indicates a difference from GFP-positive ESC clones at day 1. Statistical significance is determined by two-tailed

Student's t test. Data are represented as mean ± SEM. */#p < 0.05, **/##p < 0.01, ***p < 0.001. Source data are provided as a Source Data file.

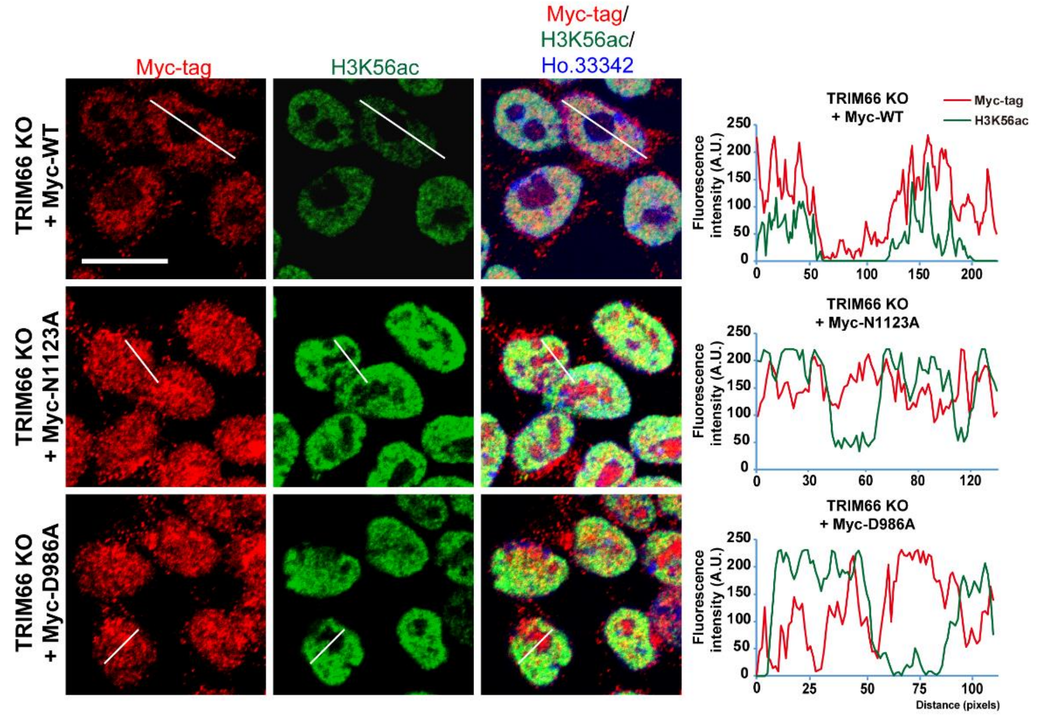

Supplementary Figure 11. TRIM66 protein is co-localized with H3K56ac in ESC nucleus. Representative immunofluorescence images of H3K56ac and Myc-tagged TRIM66 WT or mutants overexpressed in TRIM66 KO ESCs and the intensity profile of both H3K56ac and TRIM66 WT or mutants across the white line as shown in the images. Scale bar, 10 μm.

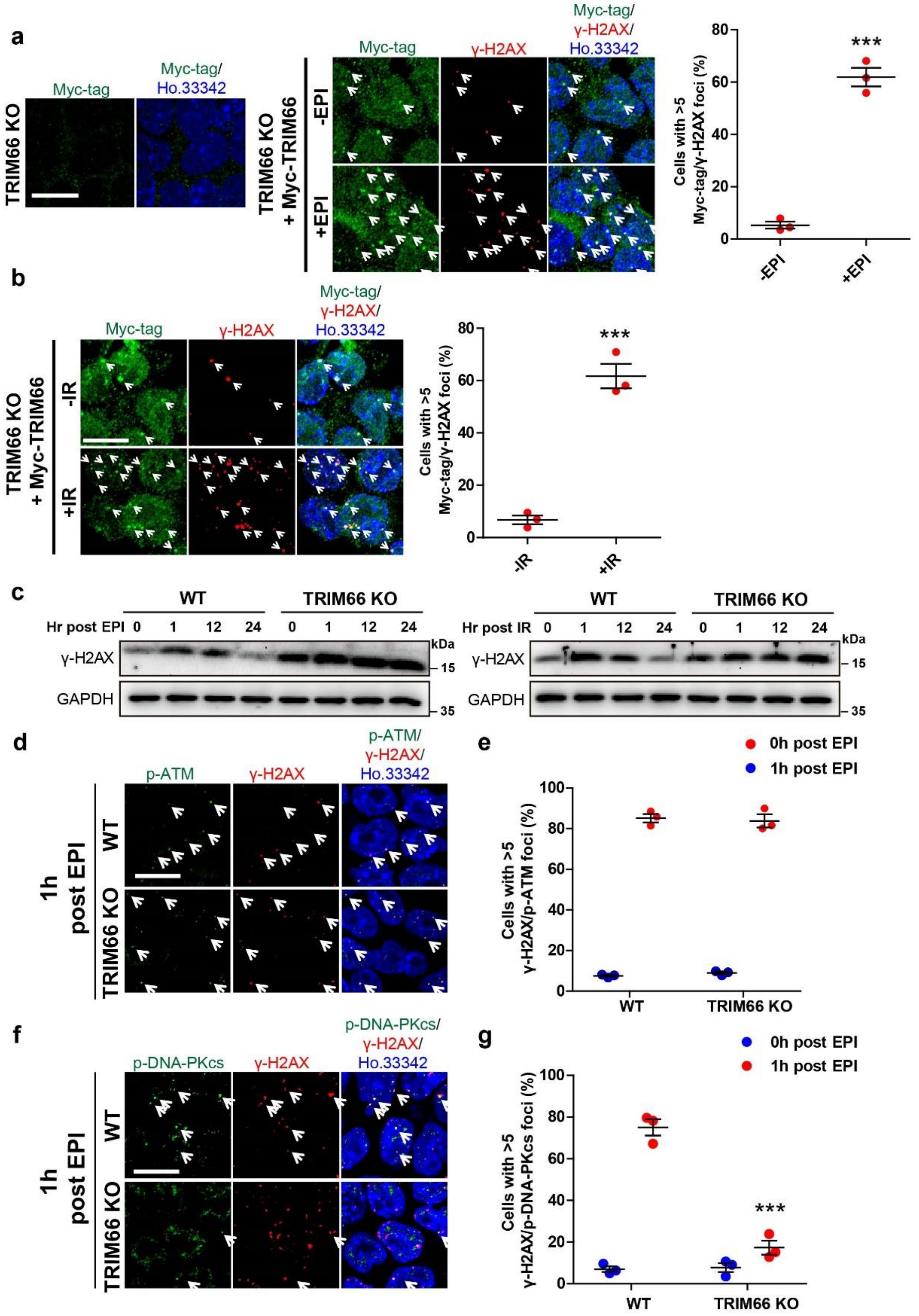

Supplementary Figure 12. TRIM66 locates at DNA damage sites and regulates DDR.

a, Representative immunofluorescence images of Myc-tag in TRIM66 KO ESCs as a negative control (left). Representative images and quantification of the co-localized γ-H2AX and Myc-tagged TRIM66 overexpressed in TRIM66 KO ESCs under the treatment with or without EPI (right). Arrowheads indicates the co-localized foci. Scale bar, 10 μm. **b,** Representative images and quantification of the co-localized γ-H2AX and Myc-tagged TRIM66 overexpressed in TRIM66 KO ESCs under the treatment with IR or without IR (right). Arrowheads indicates the co-

localized foci. Scale bar, 10 μm. **c,** WT and TRIM66 KO ESCs are treated with EPI or IR, immunoblot analysis of γ-H2AX after 0 h, 1 h, 12 h, and 24 h of recovery. **d, e,** Representative immunofluorescence images **d** and quantification **e** of the co-localized p-ATM and γ-H2AX foci in WT and TRIM66 KO ESCs after treated with EPI for 1 h. Arrowheads indicates the co-localized foci. Scale bar, 10 μm. More than 100 cells are examined in each sample. **f, g,** Representative immunofluorescence images **f** and quantification **g** of the co-localized p-DNA-PKcs and γ-H2AX foci in WT and TRIM66 KO ESCs after treated with EPI for 1 h. Arrowheads indicates the co-localized foci. Scale bar, 10 μm. More than 100 cells are examined in each sample. Data are presented as the means ± SEM. Statistical significance is determined by two-tailed Student's t test. *p < 0.05, **p < 0.01, ***p < 0.001. Source data are provided as a Source Data file.

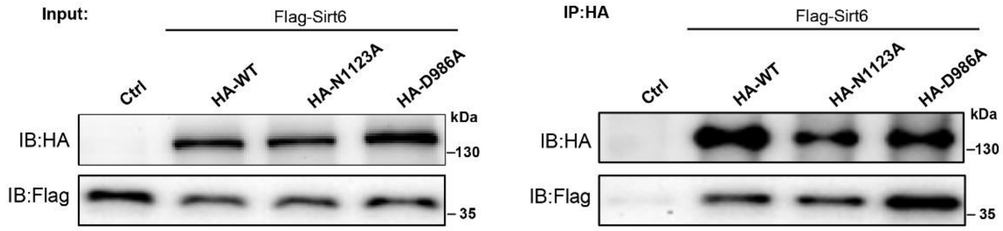

Supplementary Figure 13. Histone PTM recognize mutations of TRIM66 could associate with Sirt6.

Co-immunoprecipitation analysis of Sirt6 and the point mutations of TRIM66 in 293T cells. Source data are provided as a Source Data file.

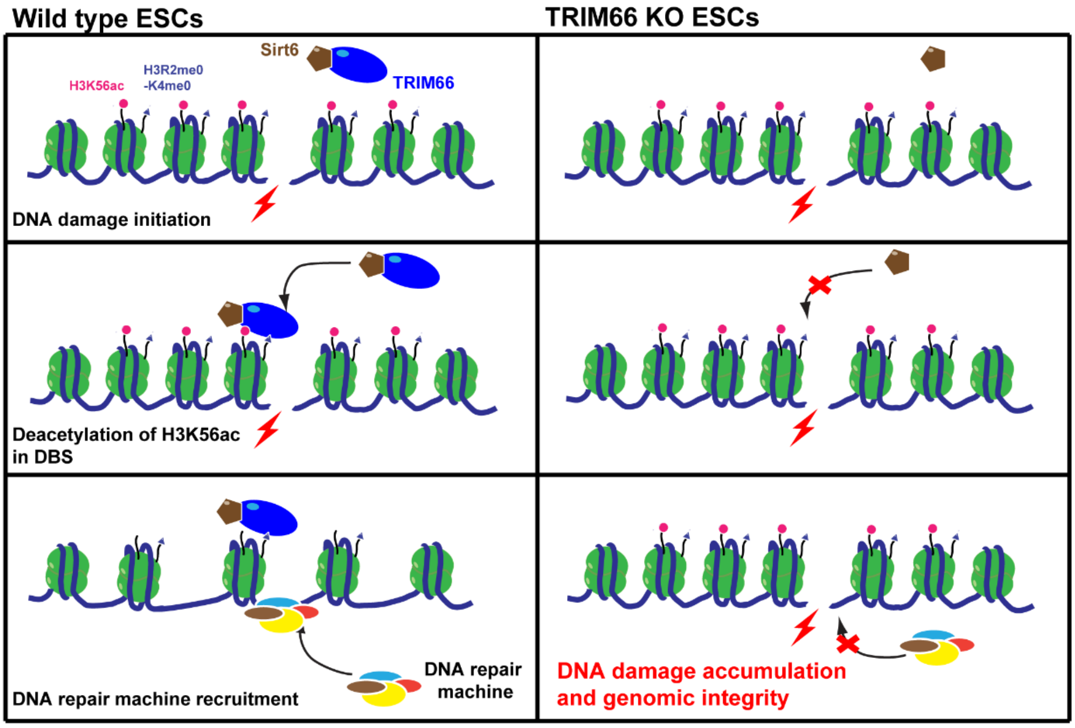

Supplementary Figure 14. Model for TRIM66 function in DDR.

TRIM66 binds to modified histone H3R2me0K4me0-K56ac and recruits Sirt6 to DBS, promoting the subsequent recruitment of DNA repair machine proteins in response to DNA lesion in ESCs.

Supplementary Tables

Supplementary Table 1. The information of the proteins and peptides that are used.

	organisms	Uniprot Entry	Mutation
TRIM66-WT$_{965-1160}$ (PHD-Bromodomain)	Homo sapiens	O15016 (Isoform 1)	None or (E968A, N969A, D971A, D986A, N1123A or D986A/N1123A)
TRIM66-MUT$_{968-1160}$ (PHD-Bromodomain)			With eight residues mutated (L1002T, C1026S, C1030S, Y1031H, M1036K, I1089T, C1135S, and V1138N)
TRIM66-GSGS$_{968-1160}$ (PHD-Bromodomain)			GSGS replace residues 1030-1040
TRIM24$_{824-1006}$ (PHD-Bromodomain)	Homo sapiens	O15164 (Isoform long)	
TRIM33$_{882-1087}$ (PHD-Bromodomain)	Homo sapiens	Q9UPN9 (Isoform α)	
ASF1a$_{1-156}$	Homo sapiens	Q9Y294	
H3$_{\text{full length}}$	Xenopus laevis	A0A310TTQ1	
H4$_{\text{full length}}$	Xenopus laevis	P62799	
H3.1 (peptide)	Homo sapiens	P68431	
H4 (peptide)	Homo sapiens	P62805	

Supplementary Table 2. ITC results.

Protein	Peptide	$\triangle H$ (kcal per mol)	$-T\triangle S$ (kcal per mol)	N	K_D μM
TRIM66-WT$_{965-1160}$	H3$_{1-12}$	-7.18±0.08	0.62	0.96±0.01	15.70±0.64
TRIM66-WT$_{965-1160}$	H3K56ac$_{48-57}$	-11.80±0.62	6.39	1.07±0.05	109.00±5.09
TRIM66-WT$_{965-1160}$	H3K56ac$_{(1-15)-(48-57)}$	-15.5±0.15	6.91	1.17±0.01	0.52±0.05
TRIM66-WT$_{965-1160}$	H3$_{48-57}$			N.D.	
TRIM66-GSGS$_{968-1160}$	H3K56ac$_{48-57}$	-10.50±0.84	5.23	1.23±0.08	127.00±9.53
TRIM66-MUT$_{968-1160}$	H3$_{1-12}$	-6.62±0.09	0.18	1.12±0.01	19.00±0.76
TRIM66-WT$_{965-1160}$	H3R2me1	-5.51±0.16	-0.13	0.91±0.01	72.40±3.27
TRIM66-WT$_{965-1160}$	H3R2me2a	-4.39±0.37	-1.00	0.98±0.04	110.00±11.90
TRIM66-WT$_{965-1160}$	H3R2me2s			[a]Weak binding	
TRIM24$_{824-1006}$	H3$^{1-12}$	-7.02±0.26	1.16	0.92±0.02	51.40±3.49
TRIM24$_{824-1006}$	H3R2me1	-7.10±0.15	1.45	0.93±0.01	72.70±2.23
TRIM24$_{824-1006}$	H3R2me2a	-7.39±0.10	1.78	0.86±0.01	76.80±1.20
TRIM24$_{824-1006}$	H3R2me2s			Weak binding	
TRIM33$_{882-1087}$	H3$^{1-12}$	-6.98±0.04	-0.49	1.06±0.00	3.39±0.08
TRIM33$_{882-1087}$	H3R2me1	-8.83±0.08	1.60	0.80±0.00	5.10±0.15
TRIM33$_{882-1087}$	H3R2me2a	-7.12±0.07	0.02	1.06±0.01	6.23±0.23
TRIM33$_{882-1087}$	H3R2me2s			Weak binding	
TRIM66-WT$_{965-1160}$	H3K4me1	-5.59±0.13	-0.37	1.10±0.01	42.80±2.45
TRIM66-WT$_{965-1160}$	H3K4me2			Weak binding	
TRIM66-WT$_{965-1160}$	H3K4me3			Weak binding	
TRIM66$_{965-1160\ E968A}$	H3$^{1-12}$	-6.40±0.10	0.32	1.04±0.01	34.80±1.41
TRIM66$_{965-1160\ N969A}$	H3$^{1-12}$	-5.18±0.22	-0.46	0.84±0.02	72.60±4.57
TRIM66$_{965-1160\ D971A}$	H3$^{1-12}$			Weak binding	
TRIM66$_{965-1160\ D986A}$	H3$^{1-12}$			Weak binding	
TRIM66$_{965-1160\ N1123A}$	H3$^{1-12}$			N.D.	

K_D values were calculated from single measurement and errors were estimated from fitting curve by MicroCal PEAQ-ITC analysis software.

[a]ITC curves cannot be fitted reliably.

N.D. no detectable binding

Supplementary Table 3. Primers used for vector construction in protein purification.

construct name	sequence
TRIM66-WT$_{968\text{-}1160}$	F: GGAATTCCATATGGAGAATGAGGACTTCTGTGCTGTTTGC
	R: CCGCTCGAGTCAGTCCTCCTGCCTTGGCTGGGCAAACCG
TRIM66-GSGS$_{968\text{-}1160}$	F: GGCTCAGGCTCAGGCCTAAGCATGTATGACCAGA
	R:GGCATTCTCACAGTCGTACTCCATCTCGGG
TRIM66-WT$_{965\text{-}1160}$-optimized	F:GGAATTCCATATGGCCCCGATTGAAAACGAAGATTTCTGC
	R:CCGCTCGAGCTAATCTTCCTGGCGCGGCTGTGCAAAACGT
TRIM66-E968A	F:ATGGCCCCGATTGCAAACGAAGATTTCTGCGCC
	R:ATGCGGGGATCCACGCGGAACCAGATCCGA
TRIM66-N969A	F:ATGGCCCCGATTGAAGCAGAAGATTTCTGCGCC
	R:ATGCGGGGATCCACGCGGAACCAGATCCGA
TRIM66-D971A	F:ATGGCCCCGATTGAAAACGAAGCATTCTGCGCC
	R:ATGCGGGGATCCACGCGGAACCAGATCCGA
TRIM66-D986A	F:GTGTTGCGCACGCTGTCCGAAAGTGTTTCA
	R:AGCAGTTCGCCGCCATTCAGACACACG
TRIM66-N1123A	F:AAATTCGCATACCCGGATAGTGAAGTGGCC
	R:TGCACAATTCCAAAACATCAGGCGCACATC
TRIM66-MUT$_{968\text{-}1160}$	F:GGAATTCCATATGGAGAACGAAGATTTTTGTGC
	R:CCGCTCGAGCTAATCTTCCTGACGCGGCTGTGCA
TRIM24$_{824\text{-}1006}$	F:GGAATTCCATATGCCCAATGAGGACTGGTGTGCAGTTT
	R:CCGCTCGAGCTATGGATAGAGGTTCTTTAGAAGTTCT
TRIM33$_{882\text{-}1087}$	F:GGAATTCCATATGGATGATGACCCAAATGAAGACTGGT
	R:CCGCTCGAGCTATGCGAAGGTCCTGTCTGAGTAGATC

Supplementary Table 4. Primers or sequences used for vector construction.

Guide RNA of Trim66	TCTGCACATACTGCAACCGC	
Doner_5' arm_PF	GAGTGGAAGGTGAAGGTATC	
Doner_5' arm_PR	CTGGAAGCAGAGAGTCCA	
Doner_3' arm_PF	GTACCAGCCCCCTACCCTGCTTC	
Doner_3' arm_PR	CTGCCAGAGAAGAAAGAAGATG	
Trim66-F1_PF	ATGGCCAGGAACTGCTCTGAG	
Trim66-F1_PR	TCAAGGATCTGTGTTACAAC	
Trim66-F2_PF	ATGGGCTCCCCTTGGAGTATC	
Trim66-F2_PR	TCAAGACAGTCTGTCCTGGC	
Trim66-F3_PF	ATGGAGGCCACCCAGGCCCC	
Trim66-F3_PR	TCACACCTGAGAGATGCTGTTG	
shTrim66-1	CATCAACTGGGCTGTCTGCAG	
shTrim66-2	GTATCAGATTCACCTGGGAGC	
TRIM66 D986A	GTTTGCCTCAATGGCGGAGAGTTACTGTGCTGT **GCC** CGCTGCCCCA	F
TRIM66 D986A	GGTGGAACACTTTGGGGCAGCG **GGC** ACAGCACAGTAACTCTCCGCC	R
TRIM66 N1123A	GCGCCTCATGTTCTGGAACTGTGCTAAGTTC **GCT** TATCCTGACTCCGA	F
TRIM66 N1123A	GCCTCTGCAACCTCGGAGTCAGGATA **AGC** GAACTTAGCACAGTTCC	R

Supplementary Table 5. Primers used in RT-qPCR assays.

Trim66	AACTGCTCTGAGTGCAAGG	F
	GGATCCCTTCTGTGCCCGTGC	R
Oct4	TCTTTCCACCAGGCCCCCGGCTC	F
	TGCGGGCGGACATGGGGAGATCC	R
Sox2	AATACCGGCCGCGGCGGAAAACCAA	F
	TTGCTCCAGCCGTTCATGTGCGCGT	R
Nanog	CAGGTGTTTGAGGGTAGCTC	F
	CGGTTCATCATGGTACAGTC	R
T	GGTGGCTTGTTCCTGGTGC	F
	GTAGGTGGGCTGGCGTTAT	R
Tuj1	TAGACCCCAGCGGCAACTAT	F
	GTTCCAGGTTCCAAGTCCACC	R
Dnmt3b	CTCGCAAGGTGTGGGCTTTTGTAAC	F
	CTGGGCATCTGTCATCTTTGCACC	R
Cxcr4	TCCAACAAGGAACCCTGCTTC	F
	TTGCCGACTATGCCAGTCAAG	R
Cdkn1a	CTGTCTTGCACTCTGGTGTCTGA	F
	CCAATCTGCGCTTGGAGTGA	R
Gadd45a	AGGCTGCCAAGCTGCTCAA	F
	AGCAGCCAGCAGGCACAGTA	R
p53	GCGTAAACGCTTCGAGATGTT	F
	TTTTTATGGCGGGAAGTAGACTG	R

Supplementary Table 6. Antibodies used in this assay.

Antibody	Company	Dilution
γ-H2AX	Abcam (ab26350)	1:1000 (IF); 1:2000 (WB)
H3K56ac	Abcam (ab71956)	1:1000 (IF); 1:2000 (WB)
GAPDH	Bioworld (ap0063)	1:3000 (WB)
HA-tag	Abcam (ab9110)	1:2000 (WB)
β-Tubulin	Invitrogen (18-0093)	1:3000 (WB)
Histone H3	Abcam (ab1791)	1:3000 (WB)
TRIM66	Abcam (ab108445)	1:500 (IF); 1:2000 (WB)
Sirt1	Millipore (04-1557)	1:2000 (WB)
Sirt6	Abcam (ab62739)	1:2000 (WB)
Flag-tag	Cell signaling (14793s)	1:2000 (WB)
Nanog	Abcam (ab80892)	1:2000 (WB)
Oct4	Abcam (ab19857)	1:2000 (WB)
Sox2	Abcam (ab59776)	1:2000 (WB)
Myc-tag	Cell signaling (2276s)	1:1000 (IF)
p-DNA-PKcs	Abcam (ab44815)	1:1000 (IF)
p-ATM	Millipore (05-740)	1:1000 (IF)
Rad51	Cell signaling (8875s)	1:1000 (IF)
CtIP	Abcam (ab38016)	1:1000 (IF)

Supplementary Notes

Supplementary Note 1. The mutation of TRIM66-MUT$_{968-1160}$.

The crystallization of the TRIM66 protein (residues$_{968-1160}$) was challenging. To overcome this problem, we mutated eight residues (TRIM66-MUT$_{968-1160}$) based on its sequence similarity with TRIM24 and TRIM33 (Supplementary Fig. 1c). The heteronuclear single quantum correlation (HSQC) NMR spectrum of TRIM66-MUT$_{968-1160}$ overlaps with that of the TRIM66-WT$_{968-1160}$ (Supplementary Fig. 1d, 1e) which showed that the introduced mutations do not result in significant structural alterations.

Supplementary Note 2. The mutation of TRIM66-GSGS$_{968-1160}$.

We created a mutant protein (termed TRIM66-GSGS$_{968-1160}$), in which a GSGS fragment replaced the linker residues 1130-1140. The HSQC NMR spectrum of this mutant protein overlapped with that of the WT protein globally (Supplementary Fig. 1e, 7a). In addition, the TRIM66-GSGS$_{968-1160}$ showed a similar binding affinity (K_D = 127.00 μM) to that of the WT (Supplementary Fig. 7b).

Supplementary Note 3. The backbone assignment of TRIM66-GSGS$_{968-1160}$.

To determine the residues types of those perturbed peaks in NMR titration, we tried to assign all the backbone NMR signals. Unfortunately, the backbone sequential assignment was unfeasible using a routine procedure due to poor quality of the $^{13}C_\beta$-correlated triple-resonance spectra. However, thanks to the newly developed NMR non-uniform sampling technique and backbone covariance LCC assignment protocol[2,3], we successfully assigned ca. 91.4% of all residues (proline excluded) (Supplementary Fig. 7d).

Supplementary Methods

The codon optimized DNA sequence of TRIM66-MUT$_{968\text{-}1160}$ and TRIM66-WT$_{965\text{-}1160}$

TRIM66-MUT$_{968\text{-}1160}$:

ATGGAGAACGAAGATTTTTGTGCCGTTTGTCTGAACGGTGGTGAACTGCT
GTGTTGTGATCGTTGTCCGAAAGTTTTTCATCTGAGCTGTCATGTTCCGGC
ACTGACCAGCTTTCCGGGTGGTGAATGGGTTTGTACCCTGTGTCGTAGCCT
GACCCAGCCGGAAATGGAATATGATAGCGAAAATGCAAGCCATAATCAG
CCGGGTAAACGTGCAAGCCCGGGTCTGAGCATGTATGATCAGAAAAAATG
TGAAAAACTGGTGCTGAGCCTGTGTTGTAATAATCTGAGCCTGCCGTTTCA
TGAACCGGTTAGCCCGCTGGCACGTCATTATTATCAGATTATTAAACGTCC
GATGGATCTGAGCACCATTCGTCGTAAACTGCAGAAAAAAGATCCGGCAC
ATTATACCACCCCGGAAGAAGTTGTTAGCGATGTTCGTCTGATGTTTTGGA
ATTGTGCAAAATTTAACTACCCGGATAGCGAAGTTGCAGAAGCAGGTCGT
AGCCTGGAAAATTTTTTTGAAGGTTGGCTGAAAGAAATCTATCCGGAAAA
ACGTTTTGCACAGCCGCGTCAGGAAGATTAG

TRIM66-WT$_{965\text{-}1160}$:

ATGGCCCCGATTGAAAACGAAGATTTCTGCGCCGTGTGTCTGAATGGCGG
CGAACTGCTGTGTTGCGATCGCTGTCCGAAAGTGTTTCATCTGAGCTGCCA
TGTTCCGGCACTGCTGAGTTTTCCGGGCGGTGAATGGGTGTGCACCCTGTG
CCGCAGCCTGACCCAGCCGGAAATGGAATATGATTGCGAAAATGCATGTT
ATAACCAGCCGGGTATGCGTGCAAGCCCGGGCCTGAGCATGTATGATCAG
AAAAAATGTGAAAAGCTGGTGCTGAGCCTGTGTTGCAATAATCTGAGTCT
GCCGTTTCATGAACCGGTGAGTCCGCTGGCACGTCATTATTATCAGATTAT
TAAGCGCCCGATGGATCTGAGTATTATTCGTCGCAAACTGCAGAAAAAAG
ATCCGGCACATTATACCACCCCGGAAGAAGTGGTTAGTGATGTGCGCCTG
ATGTTTTGGAATTGTGCAAAATTCAATTACCCGGATAGTGAAGTGGCCGA
AGCCGGTCGTTGTCTGGAAGTTTTCTTTGAAGGTTGGCTGAAAGAAATCTA
TCCGGAAAAACGTTTTGCACAGCCGCGCCAGGAAGATTAG

The purification procedure of histone H4 and H3K56ac

The cells were sonicated in buffer 20 mM Tris, pH 7.8, 500 mM NaCl, 1mM EDTA, 1 mM PMSF, and 1 mM DTT. After sonicate, the lysate was centrifuged to get inclusion body. Then the inclusion body was sonicated in 6 M GuHCl and 50 mM phosphate, pH 7.2 until the solution clear. Centrifuge the solution at room temperature to get clear supernatant. After dialyzing the supernatant against 8 M urea, 50 mM phosphate, pH 7.2, and 50-100 mM NaCl overnight in cold room, incubating the solution with SP sepharose (GE Healthcare) at room temperature for 2 h. Then get off the solution and wash column 5 times with 10 ml of 8 M urea, 50 mM phosphate, pH 7.2, and 100-150 mM NaCl. Finally, elute histone with 10 ml of 6 M GuHCl and 50 mM phosphate, pH7. 2. Further purification was achieved through high performance liquid chromatography (HPLC) using HPLC column (YMC company, lot: 12341). Freeze-dry the solution to storage the histone protein.

The recombination methods of the H3-H4-ASF1a complex *in vitro*

The freeze-dried histone protein, H3K56ac, and H4 were dissolved in unfolding buffer (7 M GuHCl, 20 mM Tris, pH7.5, and 5 mM DTT) at molar ratio of 1:1.2 and dialyzed against buffer 10 mM Tris, pH 7.5, 2 M NaCl, 1 mM EDTA, and 1 mM DTT at 4°C for 24 h. Then dialyzing histone against a lower-salt buffer 20 mM Tris, pH 8.0, 500 mM NaCl, 1 mM EDTA, and 1 mM DTT at 4°C for 12 h. The sample was further applied to a Superdex HiLoad 200 16/60 column (GE Healthcare). The eluted fractions were analyzed by SDS-PAGE. The purified proteins, ASF1a and the histone H3-H4 complex were mixed at an approximate molar ratio of 1.5:1 and dialyzed against buffer 20 mM Tris, pH 8.0, 500 mM NaCl, 1 mM EDTA, and 1 mM DTT at 4°C for 4 h. Then, the sample was applied to a Superdex HiLoad 200 16/60 column (GE Healthcare) to get H3-H4-ASF1a complex.

Supplementary References

1. Song, Z. et al. Solution Structure of the Second RRM Domain of RBM5 and Its Unusual Binding Characters for Different RNA Targets. *Biochemistry* **51**, 6667-6678 (2012).
2. Wei, Q. et al. Frontispiece: NMR Backbone Assignment of Large Proteins by Using 13Cα-Only Triple-Resonance Experiments. *Chemistry – A European Journal* **22**, 9556-9564 (2016).
3. Coggins, B.E., Werner-Allen, J.W., Yan, A. & Zhou, P. Rapid Protein Global Fold

Determination Using Ultrasparse Sampling, High-Dynamic Range Artifact Suppression, and Time-Shared NOESY. *Journal of the American Chemical Society* **134**, 18619-18630 (2012).
